# Nurse-led service delivery models in primary care: a scoping review protocol

**DOI:** 10.3399/BJGPO.2021.0194

**Published:** 2022-07-27

**Authors:** Jessica E Moulton, Nishadi Nethmini Withanage, Asvini K Subasinghe, Danielle Mazza

**Affiliations:** 1 Department of General Practice, Monash University, Notting Hill, Australia; 2 National Health and Medical Research Council SPHERE Centre of Research Excellence in Sexual and Reproductive Health for Women in Primary Care, Notting Hill, Australia

**Keywords:** nursing, midwifery, delivery of health care, telemedicine, task-sharing, primary health care, scoping review, general practice

## Abstract

**Background:**

Ensuring equitable access to health care is reliant on the strengthening of primary care services. Increasing the utilisation of task-sharing and telehealth models is one strategy to improve patient access and outcomes in primary care. This protocol details the methodology of a proposed scoping review of nurse and midwife involvement in task-sharing and telehealth models in primary care.

**Aim:**

To identify what task-sharing and telehealth models have been utilised in the primary care setting globally, and to capture the characteristics and health and economic outcomes of the models, and whether they are acceptable and feasible.

**Design & setting:**

This protocol was developed in line with the Joanna Briggs Institute (JBI) methodology for scoping reviews and reported according to the Preferred Reporting Items for Systematic Reviews and Meta-analysis Protocols (PRISMA-P).

**Method:**

Five databases (Ovid MEDLINE, Embase, PubMed, Cumulative Index to Nursing and Allied Health Literature [CINAHL] and Cochrane Library) will be searched for relevant studies published in English. Articles will be screened for inclusion in Covidence by three authors, with data extracted and synthesised using a chart designed for this review. Evidence will be mapped in both tabular and narrative forms to show characteristics, outcomes, and acceptability of the models of care.

**Conclusion:**

Understanding how nurse- and midwife-led models of care may operate is crucial to strengthening service provision in primary care. Evidence on nurse and midwife-led primary care models will be collated and synthesised to inform future models.

## How this fits in

Task-sharing models of care among primary care teams can be utilised as a means of building capacity to enhance the primary care system. While systematic reviews have been conducted exploring specific aspects of nurse-led task-sharing in primary care, a broader and comprehensive synthesis — one that captures both the characteristics of models of care and outcomes — is required to understand the full breadth of the role of nurses and midwives in task-sharing globally, in order to adapt these models to new clinical areas.

## Introduction

Strengthening primary healthcare services is crucial for ensuring equitable access to essential health care. In 2018, the Declaration of Astana called for action to strengthen primary care as a means of achieving universal health coverage through promoting multisectoral action, empowering local communities, and enhancing capacity and infrastructure to build sustainable primary care systems.^1^ Telehealth and task-sharing among primary care teams, especially pertaining to GPs and practice nurses, can be utilised as a means of building capacity to enhance the primary care system.

Improving strategies to deliver care in primary care settings is critical due to an ageing population, workforce shortages, increasing burden of complex chronic diseases, and inequitable health outcomes and access.^2^ Using task-sharing models, in which health care tasks usually completed by physicians are shared with nurses, and adopting nurse-led telehealth models of care in general practice, are opportunities to improve service provision and health outcomes. Data from a literature review on human resources optimisation in health care suggested that 25%–70% of physician tasks could be completed by non-physician health workers in advanced roles, particularly in primary care.^3^

Primary health care nurses are suitably placed to collaborate in task-sharing and telehealth models of care and support patients, while reducing the workload for physicians.^4^ The expansion of the role of nurses has arisen from a number of factors, including shortage of family doctors, health system changes, and development of new models of care,^5^ with the Netherlands, Spain, the UK, the US, and Switzerland all expanding nurse practice. Australia, Belgium, Hong Kong, Canada, Finland, France, Ireland, Poland, Japan, the Czech Republic, and New Zealand have also employed nurses in advanced roles.^5,6^

The benefits of task-sharing to both patients and staff are numerous, with efficient task-sharing shown to improve patient care^7^ and satisfaction,^8^ while increasing provider job satisfaction.^9^ One qualitative study demonstrated that teamwork and high rapport among primary care staff were important contributing factors for patient-centred care.^10^ Task-sharing not only directly addresses strategic outcomes in the Declaration of Astana, but also directly benefits staff and patient wellbeing in the primary care setting.

Telehealth in primary care, in which appointments are conducted via videoconference or telephone, provides the opportunity to increase accessibility, decrease transportation barriers, and empower patients, especially in rural and regional areas.^11^ A number of studies on telehealth services in primary care have found that telehealth is acceptable to patients, and, in some cases, *preferred* due to convenience, efficiency, privacy, and comfort.^12,13^ There has been recent increasing evidence that telehealth may exclude certain population demographics,^14,15^ and that patient preference for mode of appointment is important. However, there is still an opportunity to increase access and this has become an increasing necessity, as evidenced during the COVID-19 pandemic,^16^ especially in cases where it is the only option for a patient. As evidenced in a systematic review on the impacts and costs of telehealth services, nurse involvement is common, especially in delivering follow-up appointments,^17^ and nurse involvement has shown benefits to improving clinical indicators and reducing the need for in-clinic services.^17^

Nurses and midwives are known to empower patients and target interventions that meet the wider social determinants of health through understanding the needs of local populations.^18^ Due to training and the large sociocultural diversity of the nurse and midwifery workforce, nurse and midwife involvement in task-sharing models may aid in delivering culturally-appropriate and relevant health care, as well as reducing health inequities in primary care.^19,20^

A cross-country comparative study on task-shifting in primary care was undertaken in 2016.^21^ However, the research mostly focused on policy, finance, and educational reforms, and the extent to which task-sharing occurred.^21^ There was a lack of focus on the characteristics of models of care, and the role of nurses and midwives within these models, which is critical to informing their adaptation to other settings. One Cochrane systematic review explored the impact of nurses working as substitutes for primary care doctors on patient outcomes, processes of care, and utilisation.^22^ Despite looking at nurse substitution, a scoping review with broader inclusion criteria would allow for a wider scope of evidence. A further systematic review has been undertaken on the impact of task-sharing on the course of disease.^23^ However, although the role of nurses are discussed regarding disease outcomes,^23^ there was no discussion on specific models of care. Therefore, a scoping review is required to identify and explain different task-sharing models of care.

The aim of this scoping review is to synthesise and map current evidence on nurse and midwife involvement in task-sharing and telehealth service delivery models in primary care. This evidence will inform the feasibility and design of nurse-led models of care for provision of early medical abortion (EMA) and long-acting reversible contraception, in order to increase access to these services in the primary care setting.

## Method

The protocol has been reported in line with the PRISMA-P.^24^ The review will be conducted in accordance with the JBI methodology for scoping reviews^25^ and reported against the PRISMA Extension for Scoping Reviews (PRISMA-ScR).^26^ The JBI promotes and supports the translation of evidence into practice through the identification of feasible, appropriate, and effective healthcare practices and interventions to improve global health outcomes.^25^ The JBI Reviewers' Manual guides the planning, undertaking, and writing of scoping reviews,^25^ and was chosen as the methodological framework as the proposed review seeks to inform evidence-based practice and nurse-led models of care in general practice. In line with the JBI guidelines, the protocol will outline the research questions, inclusion criteria, search strategy, study selection, data extraction, analysis of the evidence, and dissemination of results.

### Objectives

The objective of the scoping review is to ascertain what nurse-led models of care involving task-sharing and telehealth exist in primary care.

This research is being conducted within the National Health and Medical Research Council (NHMRC) Centre of Research Excellence in Sexual and Reproductive Health (SRH) for Women in Primary Care (SPHERE).^27^ The proposal for SPHERE was developed by a multidisciplinary team of key experts and clinicians in SRH and primary care. The proposed scoping review aligns with SPHERE goals of addressing access and inequity issues in the provision of EMA services in Australia through primary care.

The Population, Concept, Context (PCC) strategy has been utilised to develop research questions.^25^ The models of care that the study seeks to assess include the involvement of nurses and/or midwives (population), in task-sharing and telehealth models of care (concept) in the primary care setting globally (context).

The review will map evidence according to the following research questions:

1. What nurse-led task-sharing and telehealth models have been utilised in the primary care setting?
2. What are the characteristics and the health and economic outcomes of the nurse-led task-sharing and telehealth models of primary care identified?

### Inclusion criteria

Articles eligible for inclusion are those published in English, as the authors do not have translation resources available. Articles that detail nurse-led models of care are eligible for the review. Articles comprising nurse-led care, in which services are run by nurses, midwives, or nurse practitioners, will be included. This may include working autonomously, managing their own caseloads, physiological assessments and care planning, initiation and delivery of treatment, monitoring of conditions and medications, and referring to specialists where appropriate.

Models of care are defined as the way health services are organised and delivered, and may also be described as a 'programme' or 'intervention'. Models of care for any health condition will be eligible for inclusion, as the authors seek an understanding of different models in primary care more broadly. Any nurse-led model of care delivered in the primary care setting will be eligible if it comprises a task-sharing or telehealth component. Task-sharing involves the safe distribution of clinical activities, tasks, and responsibilities, which would otherwise be the domain of a medical physician, to a nurse or a midwife. Telehealth is defined as a clinical consultation conducted by telephone or videoconference. Task-sharing and telehealth models of care performed by a nurse practitioner, practice nurse, midwife or equivalent will be included. For this review, primary care is defined as community health care in which the provider is the first point of contact within the healthcare system, and principle for continued care, such as general practice. Only studies from primary care settings are eligible for inclusion. Studies will be excluded if they have been conducted in settings outside of primary care or do not include a nurse-led component in the delivery of models of care.

Outcomes of interest include reported characteristics, health and economic outcomes, and any evidence for feasibility or acceptability of models. Outcomes of the review will be developed iteratively during the review process.

Literature from the following sources are eligible for inclusion:

1. Intervention studies including randomised controlled trials, cluster randomised trials and quasi-randomised trials, and pragmatic trials;
2. Observational studies;
3. Qualitative, quantitative, and mixed-methods studies that evaluate or describe different aspects of task-sharing or telehealth models of care; and
4. Grey literature, including government and non-government organisation reports or guidelines that detail task-sharing or telehealth models of care with nurse or midwife involvement.

### Search strategy

A preliminary search was conducted in August 2020 on Ovid MEDLINE (see Supplementary Table 1). The search strategy was developed in line with the PCC criteria and research questions. Key relevant articles were identified and checked for any synonymous key terms to add to the search strategy. A medical research librarian assisted in refining the strategy. The search included a combination of Medical Subject Heading (MeSH) terms and key terms for concepts including model, delivery, health service, primary health, general practice, nurse, midwife, mid-level provider, task-shifting, task-sharing, telehealth, and telemedicine. The search strategy was further refined iteratively as the authors became more familiar with the literature, and in collaboration with the SPHERE Abortion Working Group. The authors used the JBI three-step search strategy to improve search sensitivity.^25^ The following databases will be searched for eligible studies: Ovid MEDLINE, Embase, Cumulative Index to Nursing and Allied Health Literature (CINAHL), and Cochrane Library.

### Study selection

Citations identified through the search will be uploaded to Covidence, where duplicates will be removed. Studies will undergo title and abstract screening, and then full-text screening for remaining eligible articles. Three authors (JEM, NW,AKS) will independently conduct the evaluation and review of articles. If there are conflicts between any two reviewers, the third reviewer will help reach a consensus through discussion. Reference lists of included articles will be scanned for additional relevant articles. A PRISMA-ScR flow chart figure will be provided to detail the study selection process.^26^

### Data extraction

A data-charting table will be developed to record key information of the source including (but not limited to) the following: author, aims, country of origin, population, methodology, intervention type (if relevant), outcomes, and key findings relevant to the research question. The JBI template data extraction instrument^25^ will be adapted specifically for this review. The pilot form will be trialled on two or three sources by two reviewers to ensure all relevant information is extracted, which is a method favoured by other authors of scoping review conduct.^28,29^ If any additional information is required, the charting table will be updated.

### Presentation of the results

The aim of charting scoping review data is to identify, illustrate, and summarise evidence, including identifying gaps in research.^30^ Results will be presented in a number of ways to clearly and logically characterise the findings.

A narrative approach will be taken when summarising and presenting the results. Evidence will be mapped based on the key research questions, including the characteristics of the models of care, reported outcomes and impact, and any evidence on acceptability of models. When reporting outcomes of the articles, consideration will be given to the nature of primary care in included countries. Evidence mapping will also be utilised to display data. Evidence maps are a visual presentation of available evidence, and aid in identifying evidence gaps.^31^ Model of care characteristics, reported outcomes, impact, and implications will be presented in tabular form.

A table detailing each phase of the scoping review will also be included, from defining a research question to study selection, data extraction, and synthesising and disseminating findings.

### Dissemination

The findings will be disseminated to relevant key stakeholders including researchers, healthcare providers, SRH organisations, and policymakers. The review will also be disseminated in publications, conference presentations, and via the SPHERE distribution network.

## Discussion

Increasing access to primary care is a major public health priority, and thus understanding the characteristics of successful nurse and midwife-led models of care is crucial to strengthening service provision in this setting. Strengths of the review include the use of the JBI methodology for scoping reviews and the broad search. The search strategy aims to identify articles in all countries to capture the role of nurses across different health systems and innovative care in both high and low-to-middle income countries. One limitation of taking a scoping review approach is that the review will be documenting what has currently been undertaken in telehealth, but not necessarily assessing appropriateness of telehealth delivery of these services. Another limitation of the study is that the broad search strategy may elicit a high number of eligible articles across multiple settings, which may make inferences regarding commonalities more difficult. However, the authors believe that the broad search strategy will allow for a more thorough exploration of nurse-led care in line with scoping review methodology. Evidence on nurse- and midwife-led primary care models will be collated and synthesised to inform the development of such models in new clinical content areas.
